# Tumor-immune hybrid cells evade the immune response and potentiate colorectal cancer metastasis through CTLA4

**DOI:** 10.1007/s10238-024-01515-9

**Published:** 2024-11-05

**Authors:** Pariyada Tanjak, Amphun Chaiboonchoe, Thanawat Suwatthanarak, Kullanist Thanormjit, Onchira Acharayothin, Jantappapa Chanthercrob, Thammawat Parakonthun, Asada Methasate, Jared M. Fischer, Melissa H. Wong, Vitoon Chinswangwatanakul

**Affiliations:** 1 Faculty of Medicine Siriraj Hospital, Siriraj Cancer Center, Mahidol University, Bangkok, 10700 Thailand; 2 Department of Surgery, Faculty of Medicine Siriraj Hospital, Mahidol University, Bangkok, 10700 Thailand; 3 Siriraj Center of Research Excellent for Systems Pharmacology, Faculty of Medicine Siriraj Hospital, Mahidol University, Bangkok, 10700 Thailand; 4 Knight Cancer Institute, Oregon Health & Science University, Portland, OR 97201 USA; 5 Cancer Early Detection Advanced Research Center, Oregon Health & Science University, Portland , OR 97201 USA; 6 Department of Molecular and Medical Genetics, Oregon Health & Science University, Portland, OR 97239 USA; 7 Department of Cell, Developmental and Cancer Biology, Oregon Health & Science University, Portland, OR 97201 USA

**Keywords:** Circulating hybrid cells, Tumor-immune hybrid cells, CTLA4, Consensus molecular subtypes, Metastasis, Spatial transcriptomic analysis, Spatial proteomic

## Abstract

Understanding the metastatic cascade is critical for the treatment and prevention of cancer-related death. Within a tumor, immune cells have the capacity to fuse with tumor cells to generate tumor-immune hybrid cells (THCs). THCs are hypothesized to be a subset of cancer cells with the capacity to enter circulation as circulating hybrid cells (CHC) and seed metastases. To understand the mechanism of THC metastasis, we investigated CHCs in peripheral blood from patients with stage IV colorectal cancer (CRC), as well as THCs in tissues of primary colorectal cancers and their liver metastasis sites using immunofluorescence, spatial proteomic, spatial transcriptomic, molecular classification, and molecular pathway analyses. Our findings indicated a high prevalence of CHCs and THCs in patients with stage IV CRC. THCs expressed CTLA4 in primary CRC lesions and correlated with upregulation of CD68, CD4, and HLA-DR in metastatic liver lesions, which is found in the consensus molecular subtype (CMS) 1 of primary CRC tissue. Pathway analysis of these genes suggested that THCs are associated with neutrophils due to upregulation of neutrophil extracellular trap signaling (NET) and neutrophil degranulation pathways. These data provide molecular pathways for the formation of THCs suggesting fusion with neutrophils, which may facilitate extravasation and metastatic seeding.

**Supplementary Information:**

The online version contains supplementary material available at 10.1007/s10238-024-01515-9.

## Background

Distant metastases are one of the most common causes of colorectal cancer (CRC)-related mortality [1]. The mechanism of metastatic cascade, tumor cells must escape the immune system, extravasate, seed distant sites, and establish a permissive microenvironment for colonization and growth [2]. As many solid cancers exhibit leukocytic traits, it is postulated that tumor cells fuse with immune cells generating tumor-immune hybrid cells (THCs) that can evade the immune system [3, 4]. THCs are presumably the precursor to circulating hybrid cells (CHCs), which may be important for cancer plasticity and metastasis. To support this notion, THCs have been demonstrated in animal studies and confirmed in patient specimens, which enhance tumor heterogeneity and impact patient prognosis [4–8]. However, many questions also remain to be answered, such as the molecular landscape of cells after fusion, the specific markers of THCs, and their correlation with clinicopathological parameters.

THCs can be easily recognized in cell culture and animal tumor model experiments using common epithelial and leukocyte markers [3, 4]. However, THCs are much more difficult to detect in human cancers. Detection of CHCs in peripheral blood may provide a potential resource to detect THCs. CHCs are defined as cells that express both carcinoma and leukocyte proteins [4–8]. Circulating tumor cells (CTCs), which are shed from the primary or metastatic tumor, are defined by their expression of a carcinoma marker but not leukocyte markers [9, 10]. Furthermore, clusters of CTCs in cancer patients can be found as tumor-associated circulating endothelial cells, which are termed rare cells because they correlate with the features of the underlying tumor vasculature [11–13]. Several studies reported that the number of CHC is greater than CTCs in head and neck, ovarian and gastrointestinal cancers [4–7, 14]; therefore, the detection of CHC may serve as a better biomarker to inform tumor biology and metastatic cascade.

CRC is a heterogeneous disease with multiple cross-talk signaling pathways between cancer and the tumor microenvironment to drive cancer progression and invasion [15, 16]. Recently, CRC has been categorized into the four different subtypes, called consensus molecular subtype (CMS; CMS1-CMS4), and provide the best description of disease heterogeneity at the gene expression level [17]. CMS4, called mesenchymal, is enriched for microsatellite stable (MSS) tumors and is characterized by appreciable immune infiltration, intermediate between CMS1, called MSI-like, and CMS2, called canonical, and CMS3, metabolic subtypes [17, 18]. CMS4 with tumor growth factor *β* (TGF*β*)-activated stroma is a feature of poor prognosis in this subtype [17]. Up regulation of TGF*β* signaling is associated with activation of the epithelial to mesenchymal transition (EMT) pathway, and is strongly associated with immune escape in the immune tumor microenvironment [19], however, the immune system in each CMS of CRC is not obvious.

Many studies have investigated the genomic and immunologic features of immunotherapy response in MSS CRC [20, 21]. Immunotherapy has shown limited efficacy in MSS CRC; however, some CRC patients with MSS were exceptional response to neoadjuvant botensilimab (BOT), an Fc-enhanced next-generation anti–CTLA4 antibody, alongside balstilimab (BAL; an anti-PD-1 antibody) [20]. The activity of BOT/BAL regimen in patients with MSS CRC showed not only a significant increase but also a diverse array of immune cells [20]. A clinical trial of first-line durvalumab and tremelimumab with chemotherapy in RAS-mutated metastatic CRC underlined that CTLA4 expression at the tumor site was associated with better response [21]. Thus, focusing on the identification of the effective and personalized biomarker such as CHCs THCs and CTLA4 may not only increase the anticancer activity but also reduce the adverse events [22, 23]. Recognition of THCs may represent a window of opportunity to efficiently discover new drugs for the future immunotherapy. Furthermore, CMS subtype can be different in primary tumor versus the matched liver metastases [17]; therefore, we hypothesized that immune cells and THCs can be different for tumor cell immune escape in each CMS subtype.

Recently, single-cell RNA sequencing (scRNA-seq) was used to decipher cell heterogeneity, identify CTCs and CHCs, and analyze their phenotypes [24, 25]. CHCs exhibit gene expression patterns distinct from parental cells but retain the expression of critical genes of each parental cell [25]. However, scRNA-seq alone provided limited information on the functional consequences upstream or downstream of THC formation in tumor tissues [26, 27]. Currently, there are commercially available platforms for conducting spatial transcriptomics and proteomics, including the Visium platform from 10 × Genomics and the GeoMx® from NanoString Technologies. GeoMx® digital spatial profiling (DSP) is a commercialized approach that enables high-level multiplex spatial profiling of proteins and RNA in tissue samples within a defined region of interest (ROI) [28]. The commercial Visium platform can profile mRNA levels in tissues, enabling its widespread application, including spatially resolved single-cell data [29, 30]. Taken together, multiplex spatial bioimaging will be powerful in deciphering THCs in metastatic physical progression for designing efficient therapies.

In this study, we examined the diversity of CHCs, CTCs, and tumor-associated circulating endothelial cells in patients with stage IV CRC. We identified putative biological functions of THCs in primary CRC and metastatic liver tissues. Finally, we identified putative signaling pathways that are upregulated/promoted or suppressed using spatial proteomic and transcriptomic analysis.

## Materials and methods

### Patients

All patients and healthy controls were enrolled in the protocol approved by Siriraj Institutional Review Board (SIRB), a certificate of approval number Si348/2019 and Si105/2021. Informed consent was obtained from all subjects. Both groups of subjects were diagnosed by surgeons, colonoscope, and tissue biopsy pathological report. Patients with CRC stage IV who treated consecutively at the Faculty of Medicine Siriraj Hospital were enrolled, whether the patient had other diseases that can cause leukocytosis. All patients were collected peripheral blood before receiving any treatments. After surgical resection, patients were diagnosed TNM stage by using pathological (American Joint Committee on Cancer, AJCC, 7th edition) or clinical classification. Healthy control subjects who were completely done colonoscopy or diagnosed by surgeons at the Faculty of Medicine Siriraj Hospital were enrolled.

### Patient samples

All human samples, fixed formalin embedded paraffin (FFPE) tissues, and peripheral blood samples were collected and analyzed with approved protocols in accordance with the ethical requirements and regulations of the Siriraj Institutional Review Board (SIRB), a certificate of approval number Si348/2019 and Si105/2021. Peripheral blood was obtained from stage IV CRC patients (*N* = 10) before surgical resection and healthy controls (*N* = 10). For each subject, 20 ml of blood sample in a 10 ml Vacuette® K2EDTA tube, EDTA (Greiner Bio-One, Frickenhausen, Germany) and a 10 ml BD Vacutainer® sodium heparin N tube, heparin (BD, NJ, USA) was collected. A total of six primary FFPE colorectal adenocarcinoma tissues and two matched liver metastases of FFPE were collected from three CRC patients analyzed with their peripheral blood in the Siriraj Cancer Center laboratory, Faculty of Medicine Siriraj Hospital.

### Detection and quantification of CTCs and CHCs from human peripheral blood using the IsoFlux liquid biopsy system

Initially, 10 ml of peripheral whole blood samples in EDTA tube from stage IV CRC patients (*N* = 10) and healthy controls (*N* = 10) were processed using the CTCs enrichment kit from the IsoFlux liquid biopsy system (Fluxion Biosciences, CA, USA), which is based on EpCAM expression [31]. Briefly, Leucosep® tubes (Greiner, Kremsmünster, Austria) and ficoll-paque (GE Healthcare, IL, USA) were used, according to the manufacturer's instructions, to obtain the peripheral blood mononuclear cell (PBMC) fraction. The EpCAM coated magnetic bead and the IsoFlux system were used for EpCAM-positive cell selection [32]. Cells were stained with the circulating tumor cell enumeration kit (Fluxion Bio-Sciences, CA, USA), according to the manufacturer’s instructions. Briefly, immunofluorescence staining was performed using anti-cytokeratin (CK), anti-CD45 (CD45), and Hoechst 33,342 (nucleus) (IsoFlux CTC Enumeration Kit; Fluxion Bio-Sciences, CA, USA). The stained cells were mounted in multiwell plates with sensoplate glass bottom (Greiner Bio-One, Frickenhausen, Germany) for imaging. Imaging was performed using an inverted epifluorescence motorized microscope (Cytation1 imaging reader; Biotex, VT, USA). Automate quantification was performed for whole sample regions by imaging software: Gen5 3.08 (Biotex, VT, USA) with individuals blinded to the clinical status of the patients or healthy controls. CTCs were identified as those with an intact nucleated cell showing CK+/CD45− [32]. CHCs were identified as those with an intact nucleated cell showing CK+/CD45+.

### Tumor-associated circulating endothelial cells (rare cells) isolation and analysis

For rare cell detection, 10 ml of blood samples in a heparin tube from patients with stage 4 CRC (*N* = 10) and healthy controls (*N* = 10) were sent to X-Zell (X-Zell, Bangkok, Thailand) within 12 h. The samples were processed according to the X-Zell procedures [33]. Briefly, each sample was subjected to red blood cell lysis and CD45-based high flow magnetic white blood cell depletion. The remaining cells were subjected to multiplexed cryo-immunostaining with antibodies directed against CD31 (mouse IgG1 with Alexa Fluor594, WM59; Biolegend, CA, USA), CD34 (mouse IgG1 with Brilliant Violet421, 581; BD Bioscience, CA, USA), CD45 (mouse IgG1 with Pacific Orange, HI30; EXBIO, Vestec, Czech Republic), Vimentin (rabbit IgG1 with Alexa Fluor488, EPR3776; Abcam, Cambridge, UK), pan-Cytokeratin (mouse IgG1 with Pacific Blue, C-11; Abcam, Cambridge, UK), and EpCAM (mouse IgG1 with Pacific Blue, VU-1D9 (CD326); EXBIO, Vestec, Czech Republic). The company analyzed and classified atypical cells.

### The nCounter® analysis and molecular classification

#### RNA extraction and the nCounter® analysis

Total RNA was extracted from two sectioned FFPE tissues (5 μm thickness) using a high purity FFPE RNA isolation kit (Roche Diagnostics, IN, USA), strictly according to the manufacturer’s instructions. The nCounter® analysis system was used to perform the assay (Nanostring Technologies, WA, USA). A pan-cancer progression panel kit was used to measure the expression of 770 genes. The raw counts of each target gene were normalized by the geometric mean counts of 11 housekeeping genes (HRNP1, RPL27, RPL9, RPL6, RPL30, OAZ1, PTMA, RPS29, UBC, RPS12 and RPS16) and spiked controls. A threshold count value equal to 20 was used for background thresholding and normalizing the samples for differences in hybridization.

#### Classification of subtypes of CRC based on deep learning

A gene expression data set from Siriraj Hospital's CRC cohort was logarithm transformed and converted from genetic information to functional spectra associated with biological pathway activities. Subsequently, a DeepCC model (DeepCC R package version 0.1.1), containing a trained artificial neural network, was performed to extract advantageous features and classify the Siriraj hospital gene expression data into four CMS classes, CMS1, CMS2, CMS3, and CMS4 [34]

### In situ detection of CTCs and CHC from human peripheral blood using spatial proteomic analysis

PBMC from two stage IV CRC patients and two healthy controls were isolated from whole blood samples in a heparin tube using ficoll-paque (GE Healthcare, IL, USA) following the manufacturer's instructions. Briefly, whole blood was first diluted with Dulbecco's phosphate-buffered saline (DPBS; Thermo Fisher Scientific, MA, USA), overlaid on ficoll-paque, and then centrifuged at 800 × g for 20 min without brake. After centrifugation, the mononuclear cells at the interface were transferred to a new tube, diluted with DPBS, and pelleted at 800 × g for 10 min. The cells were resuspended with FACS buffer (Thermo Fisher Scientific, MA, USA). Cells then adhered to poly-d-lysine-coated slides (Thermo Fisher Scientific, MA, USA) through incubation at 37 °C for 15 min, permeabilized with Triton-X (Sigma-Aldrich, MO, USA), and fixed with 4% paraformaldehyde (PFA; Thermo Fisher Scientific, MA, USA) [4]. Before staining, slides were made with 1X citrate buffer pH 6.0 (Sigma-Aldrich, MO, USA) at high pressure and temperature for 15 min. Morphological markers included Syto13 for nuclei, CD45 for immune cells, Pan-CK (CK) for adenocarcinoma cells (GeoMx® morphology markers; NanoString Technologies, WA, USA) and EpCAM (mouse IgG2 with Alexa Fluor647, 9C4; Biolegend, CA, USA). for the epithelial cell adhesion molecule. The slides were scanned and imaged using a GeoMx® instrument (NanoString Technologies, WA, USA). CHCs were identified as those with an intact nucleated cell showing CK+/CD45+.

### Exploring THC in patient FFPE tissues using spatial proteomic analysis

5-μm thick FFPE sections from three CRC patients (six FFPE primary colorectal adenocarcinoma tissues and two matched FFPE liver metastases) were strictly prepared for DSP using manual instruction from the GeoMx instrument and the GeoMx immune cell profiling panel kit with 24 proteins (NanoString Technologies, WA, USA). Briefly, FFPEs were deparaffinized by incubating slides in (R)-(+)-Limonene (Sigma-Aldrich, MO, USA), and rehydrated with various concentrations of ethanol. Antigen recovery was performed with 1X citrate buffer pH 6.0 (Sigma-Aldrich, MO, USA) at high pressure and temperature for 15 min. Morphological markers included Syto13, CD45, CK (GeoMx® morphology markers; NanoString Technologies, WA, USA) and EpCAM(mouse IgG2 with Alexa Fluor647, 9C4; Biolegend, CA, USA).

The stained slides were loaded onto a GeoMx® instrument (NanoString Technologies, WA, USA) and scanned before the regions of interest (ROI) (approximately 20 nuclei/ ROI) were selected. The four colors of the morphological marker demarcate the regions of THC, tumor-immune cells, epithelial cells, and stromal cells. UV illumination was performed, and oligonucleotides were released. The photocleaved oligonucleotides released were collected using a microcapillary tube inspiration robotic system and transferred to a 96-microwell plate. The barcodes were counted in the nCounter® analysis system using standard procedures (NanoString Technologies, WA, USA). Normalized counts were calculated using three housekeeping proteins: GAPDH, HISTONE H3 and S6.

### Spatial transcriptomic analysis of THC in FFPE tissues at the gene expression level (Visium CytAssist)

For spatial transcriptomic construction and sequencing, FFPE sections were obtained from two CRC patients (two FFPE primary colorectal adenocarcinoma tissues and two matched FFPE liver metastases). FFPE samples that passed the RNA quality control (DV200 > 50%) were strictly prepared according to the Visium CytAssist spatial gene expression for the FFPE tissue preparation guide (CG000518, 10X Genomics, CA, USA). The library preparation was strictly performed according to the Demonstrated Protocol (CG000520, 10X Genomics, CA, USA) and proceeded with the Visium CytAssist Spatial Gene Expression for FFPE-Tissue Preparation Guide (CG000495, 10X Genomics, CA, USA) by our laboratory at Siriraj Cancer Center, Siriraj Hospital Faculty of Medicine, Mahidol University. Libraries were sequenced by Novogene Co., Ltd. (Singapore) using a NovaSeq 6000 platform (Illumina, CA, USA). For data analysis, Space Ranger 2.1.0 May 2023 (10X Genomics, CA, USA) and GRCh38-2020-A reference were used to process FASTQ files. Downstream analyzes were performed using Loupe Browser 7.0 (10X genomics, CA, USA). The *p* value reported here has been given by the Wilcoxon’s test and adjusted for multiple tests via the Benjamini–Hochberg procedure.

### Pathway analysis

Ingenuity Pathway Analysis Software (IPA 41280214 and 41,280,202, Ingenuity® Systems, https://digitalinsights.qiagen.com/, accessed on February 19, 2024) was used to examine the biological pathways. The IPA software (IPA 412480214 and 41,280,202) uses a manually curated database that contains information from several reputable sources, including published journal articles and gene annotation databases. Fisher’s exact test was used to calculate the probabilities between the input gene set and the pathway.

### Statistical analysis

The analyzes were performed using Prism 9 software (GraphPad Software, Inc., CA, USA). The Mann–Whitney U test or the T test was used to compare whether there was a difference in the dependent variable between the two independent groups. The T test or Chi-squared was performed to test the statistical significance of clinical characteristics. Statistical significance was established at *p* value < 0.05.

## Results

### Detection of CTCs, CHCs, and rare cells in peripheral blood

Before detection of THC in tissues, we examined the number of CTCs, CHCs, and rare cells in peripheral blood of ten enrolled patients with stage IV CRC (patient group) collected before resection. Furthermore, the number of cell counts was compared with those of ten healthy volunteers (healthy control). Table 1 reports the clinical characteristics of each group in this study. The results showed that total white blood cell counts were significantly higher in the patient group compared to healthy control patients (*p* value = 0.006).

**Table 1:** Clinical characteristics and demographics

	Patient group Number, *N* = 10 *N* (%)	Healthy control group Number, *N* = 10 *N* (%)	*P* value
Age, years; median [min–max]	63.5 [54–71]	44.5 [26–69]	0.11
White blood cells, cells/ul; median [min–max]	8155 [6410–10260]	6340 [5220–8930]	0.006*
CEA, ng/ml; median [min–max]	252.8 [7.66–18784]		
Sex			0.136
Male	7 (70)	4 (40)	
Female	3 (30)	6 (60)	
Moderately differentiated adenocarcinoma	10 (100)		
Stage IV (TNM stages) colorectal cancer	10 (100)		
Clinical T classification			
T4b	1 (10)		
Pathological T classification			
T3	5 (50)		
T4	1 (10)		
T4a	2 (20)		
T4b	1 (10)		
Clinical N Classification			
N1	1 (10)		
Pathological N classification			
N0	1 (10)		
N1b	3 (30)		
N2a	1 (10)		
N2b	4 (40)		
Liver metastasis	9 (90)		
Lung metastasis	1 (10)		
*KRAS* mutation status			
Wild type	5 (50)		
Mutation	2 (20)		
ND	3 (30)		
*NRAS* mutation status			
Wild type	5 (50)		
Mutation	–		
ND	5 (50)		
*BRAF* mutation status			
Wild type	5 (50)		
Mutation	–		
ND	5 (50)		
*PIK3CA* mutation status			
Wild type	3 (30)		
Mutation	–		
ND	7 (70)		
*HER2* mutation status			
Wild type	3 (30)		
Mutation	–		
ND	7 (70)		

*ND* not detected, *CEA* carcinoembryonic antigen; *The T test* or Chi-squared was performed to test the statistical significance of clinical characteristics; *, *p* value < 0.05

**Fig. 1:**
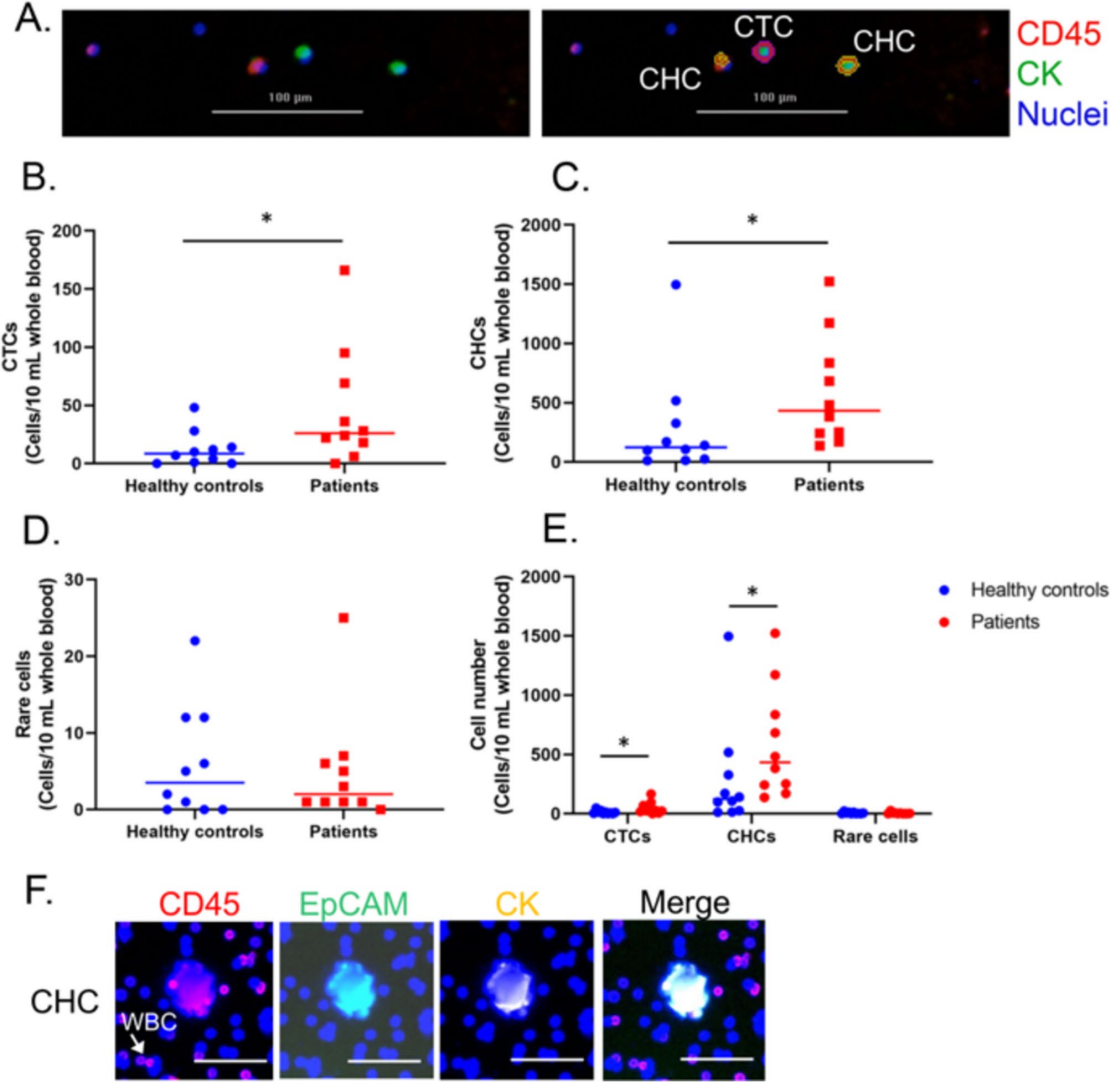
Number of cell counts in the peripheral blood of normal volunteers and patients with stage IV CRC. **A**. Representative images of typical circulating tumor cells (CTCs, CK+/CD45−) and circulating hybrid cells (CHCs, CK+/CD45+ isolated by the IsoFlux system (right panel) and counted by imaging software: Gen5 3.08 (left panel); scale bar, 100 μm** B** the number of circulating tumor cells (CTCs, CK+/CD45−) *p* value = 0.045 **C** the number of circulating hybrid cells (CHCs, CK+/CD45+) *p* value= 0.029 **D** the number of tumor-associated circulating endothelial cells (rare cells) *p* value= 0.979; **E** the number of CTC, CHC and rare cells **F** representative images of CHC (CK+/ EpCAM+/CD45+) detected by a GeoMx® instrument; scale bars, 50 μm. The Mann–Whitney U test or the T test was used to compare whether there was a difference in the dependent variable between the two independent groups. Statistical significance was established at *p* value < 0.05

After immunofluorescence staining, Fig. 1A showed that the CK and CD45 marker can identify CTCs (CK+/CD45-) and CHCs (CK+/CD45+. We found that the mean number of CTCs and CHCs showed statistical significance (*p* value = 0.045 and 0.029, respectively) in peripheral blood from patients with stage IV CRC compared to healthy volunteers. The number of CHCs (range 137–1522) was higher than the number of CTCs (range 0–136) and rare cells (range 0–25).

To investigate THC in primary CRC and metastatic liver tissues by spatial proteomic analysis, we added a more specific marker, EpCAM, to identify CHCs. The results showed that the CK, CD45, and EpCAM markers were able to identify CHC using DSP; therefore, these markers were next used to evaluate THC (CK+/EpCAM+/CD45+ in tissues.

### Spatial proteomic profiles of THCs in primary CRC and liver metastasis

**Fig. 2:**
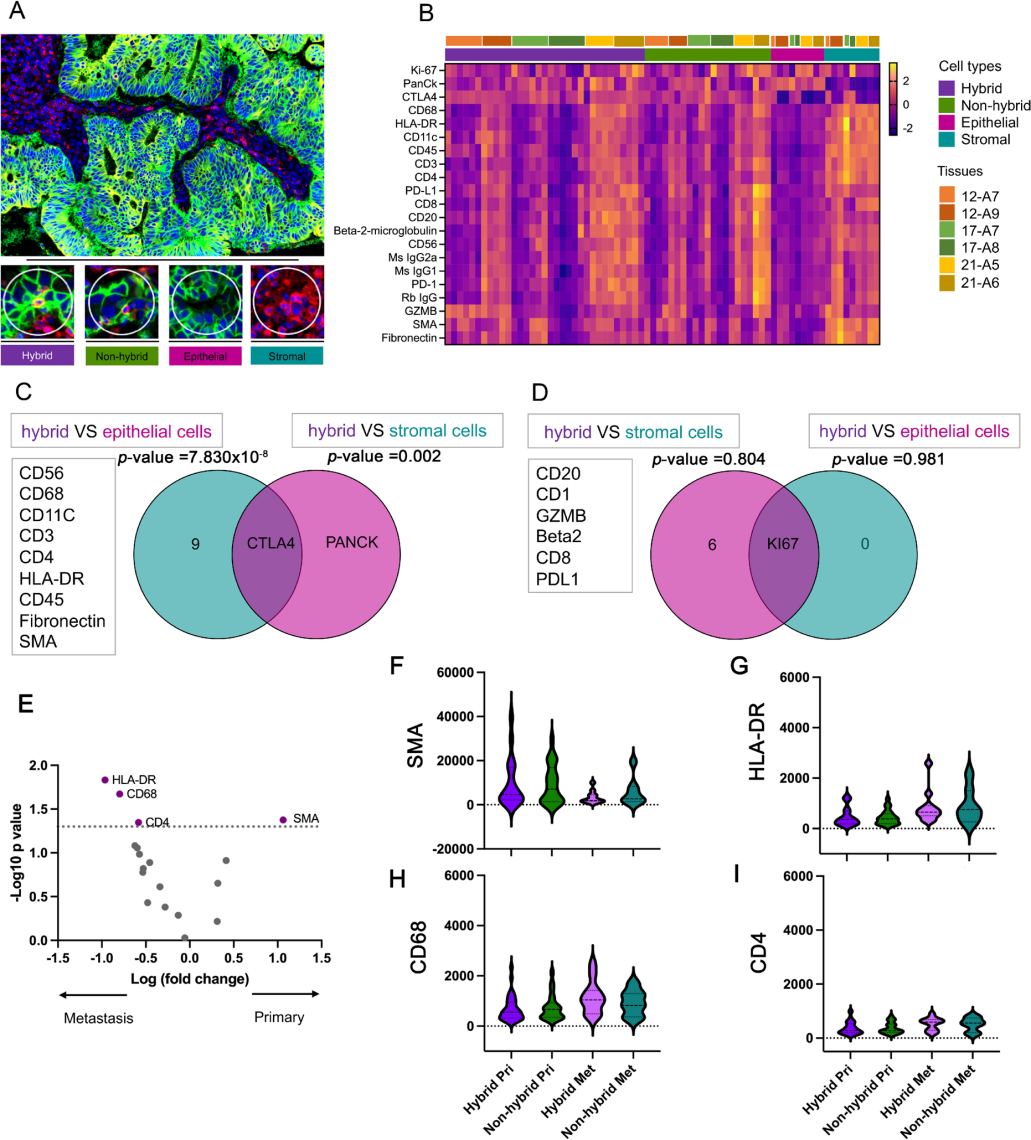
Differential protein expression of THC (hybrid cells) in primary CRC and liver metastases. (**A**) Representative image of primary CRC tissues stained by immunofluorescence with selected ROIs, hybrid cells, tumor-immune cells (non-hybrid cells), epithelial cells and stromal cells; scale bars 500 and 150 μm (**B**) Heatmap for 21 proteins (rows) from the GeoMx immune cell profiling panel are clustered by downregulation (pink) and upregulation (yellow) among hybrid cells, non-hybrid cells, epithelial cells, and stromal cells (column). Six FFPE samples from three patients 12, 17 and 21 were also clustered (column) (**C**) Venn diagram showing contrast, CTLA4 upregulation in hybrid cells (*p* value = 7.830 × 10^–8^ and 0.002 for comparing to epithelial and stromal cells, respectively). (**D**) Venn diagram showing upregulation of KI67 in hybrid cells without significant difference from epithelial cells and stromal cells (*p* value = 0.804 and 0.981 for comparing to epithelial and stromal cells, respectively). (**E**) Volcano plot of 18 proteins comparing hybrid cells between primary CRC and liver metastatic tissues (**F**) Violin plot of SMA (**G**) HLA-DR (**H**) CD68 (**I**) CD4 hybrid cells and non-hybrid cells for primary CRC and liver metastasis tissues. Volcano plots were created with a log2 fold change and an adjusted *p* value at 0.05 for cut-off. The* p* value were determined by Mann–Whitney U test or the T test

To examine the biological function of THCs, we compared the THC region with the regions of tumor immune cells, epithelial cells and stromal cells, in primary CRC (Fig. 2A). THC region was defined as the area contained THC and other cells which was approximately 20 nuclei and 2000 surface area. We found that the expression profiles of 21 THC proteins in primary CRC showed region-specific expression patterns highly expressed in stromal cell regions (Fig. 2B). We found that CD3 was significantly down-regulated in THC regions compared to tumor-immune cells (*p* value = 0.049). To identify the molecular structure of THCs, we determined the varied upregulated proteins in THCs compared to epithelial and stromal cells. We found that CTLA4 was significantly upregulated in THC regions (Fig. 2C); *p* value = 7.830 × 10^–8^ and 0.002 for comparing to epithelial and stromal cells, respectively. The THCs showed that KI67 was the same as in the regions of epithelial cells and stromal cells without significant differences (Fig. 2D); *p* value = 0.804 and 0.981 for comparing to epithelial and stromal cells, respectively. Together, CTLA4 may be a biological characteristic of THCs; while, CD3 proteins may represent immune cells (non-hybrid cells) in tumor tissues.

After investigating the biological characteristic of THC in tissues of primary CRC, we determined the characteristics of THCs between primary CRC and liver metastasis. We found that the HLA-DR, CD68, and CD4 proteins were upregulated in the THC region of liver metastasis (Fig. 2E). The SMA protein was significantly overexpressed in the THC region of primary CRC. To characterize the biological characteristics of THC between primary CRC and liver metastasis, we also compared the SMA, HLA-DR, CD68 and CD4 proteins of THC and tumor-immune cell regions between primary CRC and liver metastasis, as shown in Fig. 2F–I. The results showed that tumor-immune cell regions trended toward expressed SMA, HLA-DR, CD68 and CD4 proteins similarly to the THC regions.

### Spatial transcriptomic profiles of THCs and tumor-immune cells between primary CRC and matched liver metastases

**Fig. 3:**
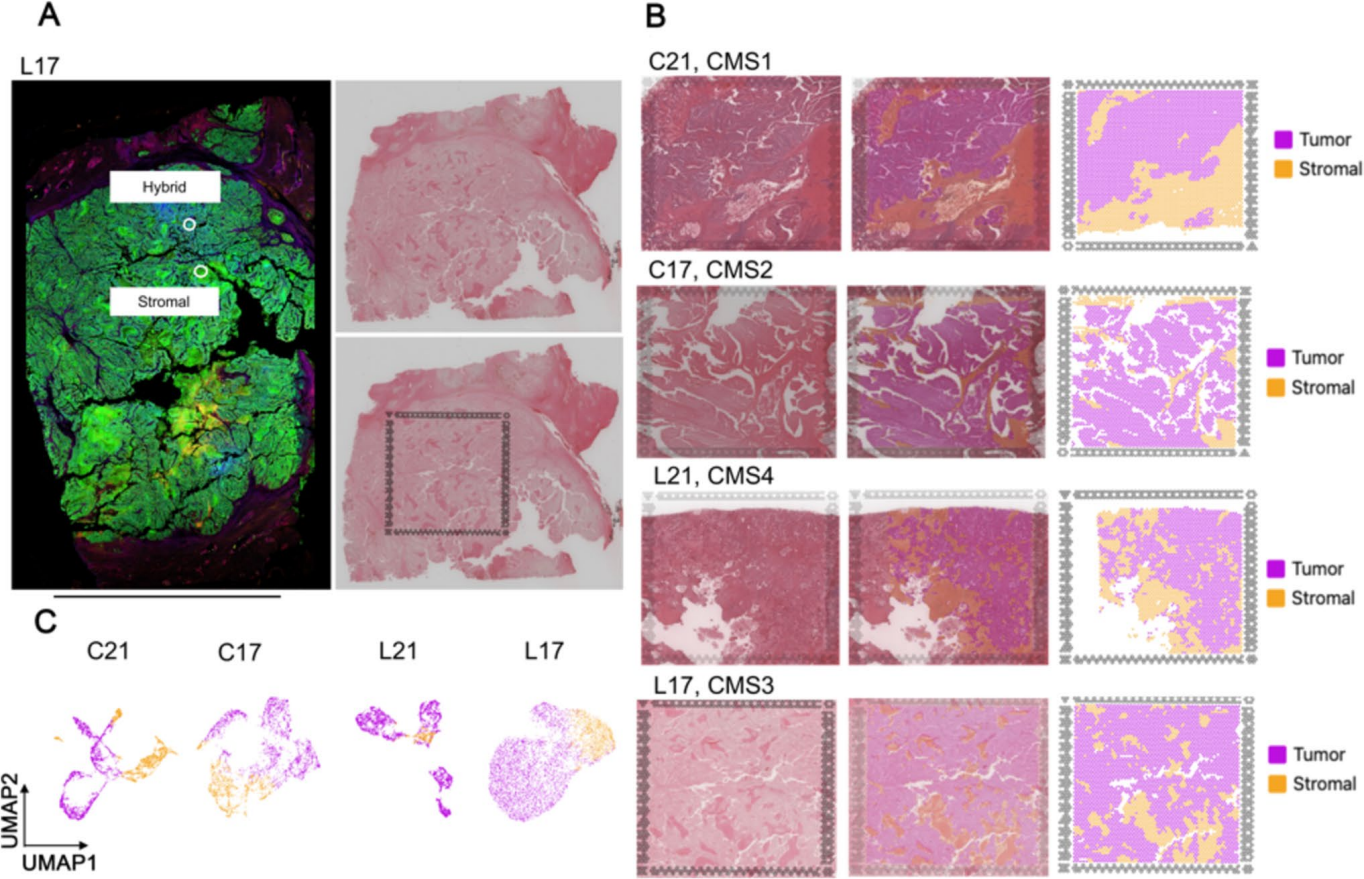
Transcriptome profiles and molecular subtypes of primary and liver metastatic tumors of CRC. (**A**) Representative image of the spatial transcriptomic analysis, covering some ROIs of the spatial proteomic analysis; scale bar 10 mm; (**B**) Overview of the spatial transcriptomic sections. H&E staining of spatial transcriptomic sections (left). Identification of the tumor and stromal area of each section (middle). Spatial cluster distribution of each section (right); analysis size 6.5 × 6.5 mm (**C**) Feature plots showing cluster of tumor and stromal areas of each section

To comprehensively analyze the prognostic effect of THCs between primary CRC tissues and liver metastatic tissues derived from CRC, we analyzed four tissue samples from two patients (21 and 17) including two primary CRC tissues (C21 and C17) and their matched liver metastatic tissues (L21 and L17). We investigated spatial transcriptomic data, including some spatial proteomic ROI (Fig. 3A). All samples were identified by CMS using their gene expression profiles. The results showed that C21, C17, L21, and L17 were classified as CMS1, CMS2, CMS4 and CMS3, respectively. The cluster of tumor and stromal areas were identified by hematoxylin and eosin (H&E) staining and gene expression profiles of each sample by Visium CytAssist (Fig. 3B). The uniform model approximation and projection (UMAP) analysis indicated the tumor and stromal areas of each sample (Fig. 3C).

### THCs and tumor immune cells between primary CRC and matched liver metastases

**Fig. 4:**
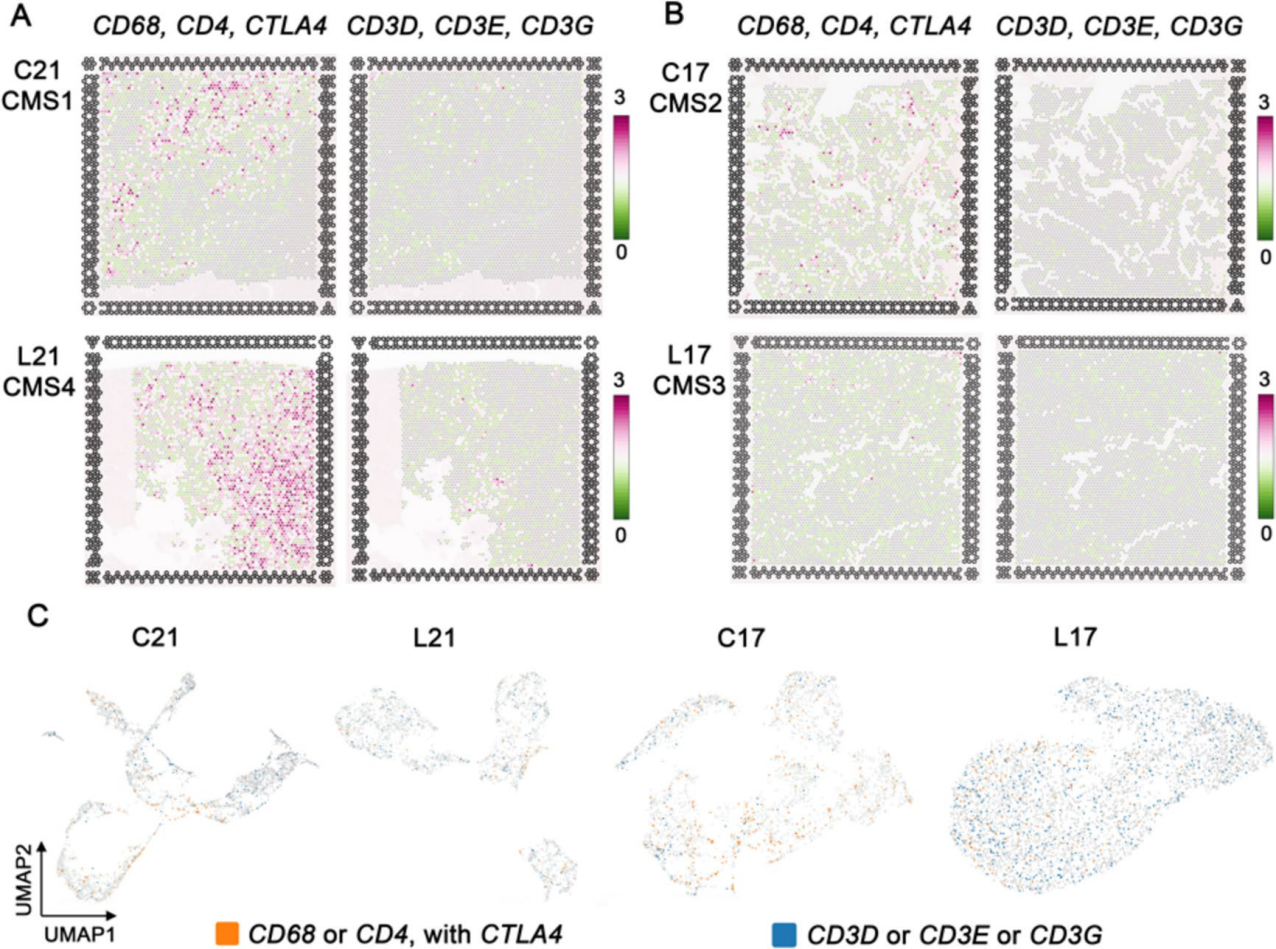
Distribution of the gene markers THC and tumor-immune cells. The maximum expression value (log2-transformed UMI counts) of *CD68*, *CD4,* and *CTLA4*, and *CD3D*, *CD3E* and *CD3G* (**A**) In patient 21; primary CRC classified as CMS4, C21 (above); liver metastasis classified as CMS4, L21 (below). (**B**) In patient 17; primary CRC classified as CMS2, C17 (above); liver metastasis classified as CMS3, L17 (below). (**C**) Feature plot of THC (*CD68* or *CD4* with *CTLA4*), and tumor-immune cells (*CD3D* or *CD3E* or *CD3G*) of each section; analysis size 6.5 × 6.5 mm

In the aforementioned results, we found that CTLA4 protein is a marker of THC regions in primary CRC that may associate with CD68 and CD4 proteins in liver metastatic of CRC. On the contrary, CD3 proteins may be a marker of tumor immune cells in primary CRC. To further explore the characteristic and prognostic value of THCs in primary CRC and in liver metastases, we investigated the distribution of the *CD68*, *CD4* and *CTLA4* genes and the distribution of the *CD3D*, *CD3G,* and *CD3E* genes in each tissue section. Spatial transcriptomic analysis showed that the infiltration of *CD68*, *CD4,* and *CTLA4* genes was higher in patient 21 (Fig. 4A) in which liver metastasis (CMS4) and primary CRC sites (CMS1) was observed compared to the infiltration of these genes in patient 17 (Fig. 4B) in liver metastasis (CMS3) of CRC (CMS2).

Then we used Loupe Browser 7.0 to group spots with gene characteristics (log2 count > 0) of *CD68* or *CD4* with *CTLA4* as THC, and spots with gene characteristics of *CD3D* or *CD3E* or *CD3G* as tumor immune cells. The feature spot showed highly arranged THCs around the tumor area (Fig. 4C).

### Pathway of THCs in the primary and liver metastatic tumors of CRC

To identify significant canonical pathways (*p* value < 0.05 or -log(*p* value) > 1.3) between THCs and tumor-immune cells, in the primary and liver metastatic tumors of CRC, we analyzed differentially expressed gene (DEG) between spots with a gene feature of THC and spots with a gene feature of tumor-immune cells using Loupe Browser 7.0 and IPA. We found that the *CD68*, *CD4*, and *CTLA4* genes are significantly upregulated in spots defined as THC for all tissues in the section. (in section C21, L21, C17 and L17, for *CD68 p* value = 1.65 × 10^–41^, 8.70 × 10^–45^, 7.96 × 10^–22^, 2.10 × 10^–34^, for *CD4 p*-value = 6.44 × 10^–28^, 5.52 × 10^–25^, 1.26 × 10^–8^, 7.65 × 10^–35^, and for *CTLA4 p*-value = 2.82 × 10^–19^, 1.58 × 10^–11^, 6.65 × 10^–16^, 6.71 × 10^–24^, respectively) (Supplementary File), therefore, the clustered spots with those genes could be represented as THC in CRC and metastasis of the matched liver. For patient 21, we found 167 and 60 significant DEG for THC spots in primary CRC and liver metastasis tissues, respectively, however, the *CD68*, *CD4* and *CTLA4* genes were only found as significant DEG for THC spots in primary CRC and liver metastasis of patient 17.

**Fig. 5:**
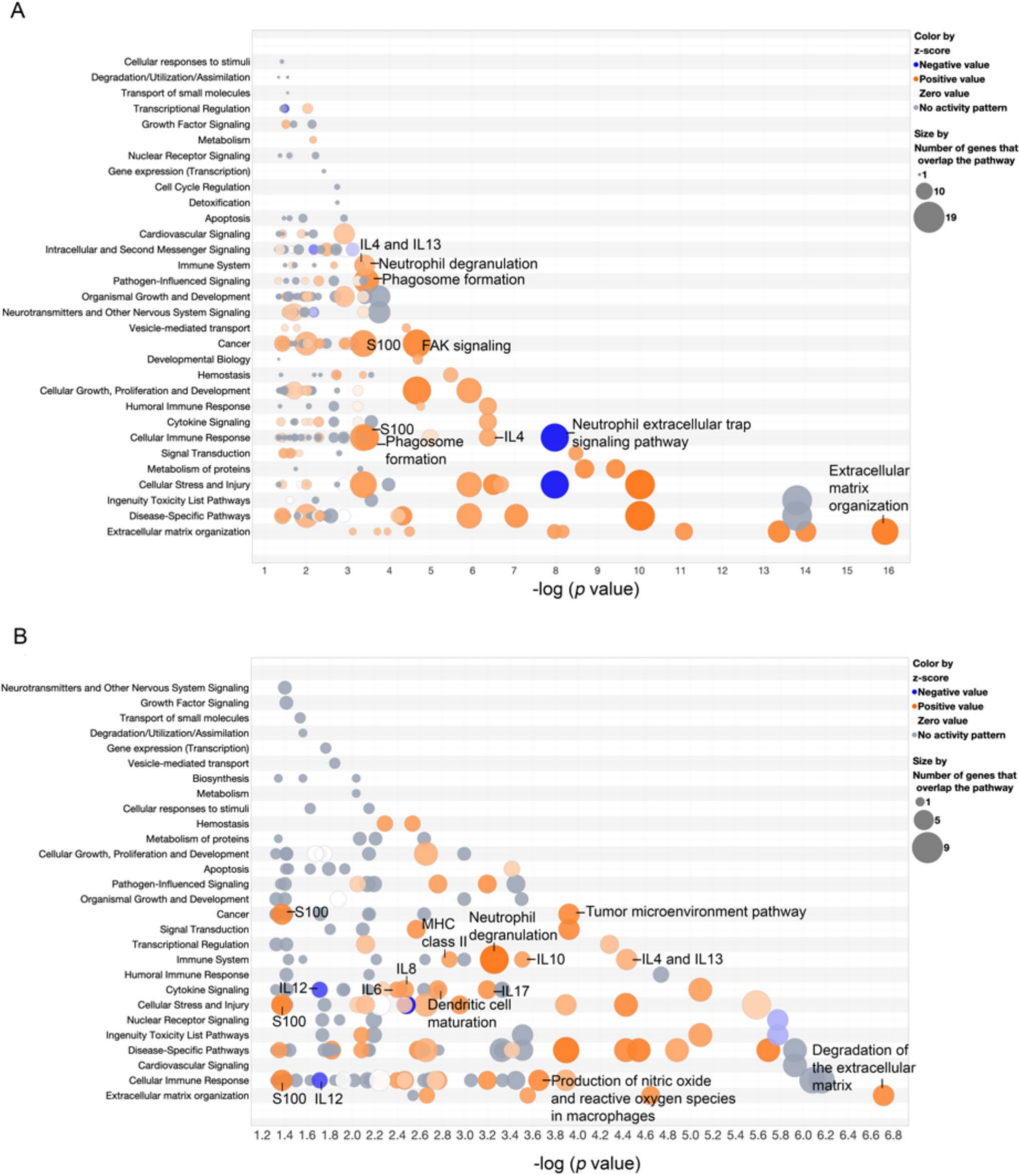
Bubble diagram of the canonical pathways from the DEG analysis of IPA of THC spots (*p* value < 0.05). The Z scores reported whether a pathway was activated: orange (z-score > 0) or inhibited: blue (z-score < 0) of the THC spots compared to the tumor immunoglobulin cell spots in patient 21 (**A**) primary colorectal cancer section (**B**) liver metastasis section. The *p* value were determined by using Fisher’s exact test

After significant DEGs were imported to the IPA, we analyzed the IPA pathway analysis of THC spots for primary CRC (Fig. 5A, Supplementary File) and for liver metastasis (Fig. 5B, Supplementary File) of patient 21. The results showed that DEGs of THC spots in primary CRC and liver metastases were enriched in activating many critical pathways associated with tumor progression, such as immune system, cancer, cellular immune response, cytokine signaling, and extracellular matrix organization signaling. Interestingly, most of the THC spots’ pathways were involved with myeloid cells, which are especially neutrophils.

## Discussion

In this study, we have shown that primary CRC might exploit myeloid and lymphocytes with CTLA4 to facilitate CRC to liver metastases. Currently, there is a wealth of accumulated data to support the hypothesis that THCs are capable of evading the host immune system and progressing to invasive carcinoma [29]. However, the upstream or downstream functional consequences of THCs in tumor tissues are still missing. Our study tried to close the gaps by elucidating and explaining the molecular pathways and the cell–cell complex between THC and cancer cells.

Our finding indicated that CHCs were found significantly in patients with CRC with liver metastasis outcome, more than in healthy donors. Additionally, lymphocyte cells in patients with CRC have been observed to be higher than those in healthy controls. Chronic inflammation is a common and important contributor to the malignant transformation of many types of solid cancer types, including cholangiocarcinoma, colorectal cancer, lung cancer, and liver cancer [35–38]. On the other hand, inflammation has recently reemerged as a paramount target in cancer therapy. Therefore, a significant understanding of immune cells and THCs may benefit for successful immunotherapy in advance cancers.

Here, we reported that CTLA4 exhibits distinct characteristics in THC regions in primary CRC. This result suggests that THC may express CTLA4 or directly increase tumor cells with CTLA4. CTLA4 plays a role in inhibitory receptors (checkpoints) that limit autoreactivity and T cell overactivation [39], also known as noninflamed tumor or cold tumor [34]. Previously, similar results were identified in the peripheral blood of patients with advanced stage CRC [40]. We suggest that CTLA4 may play an important role in the mechanism of immune evasion and metastasis of CHC and THC. Moreover, regions of non-hybrid cells show significant upregulation of CD3. This result shows a differentiation of the cell–cell complex between THC within tumors and between immune cells within tumors. CD3 has been reported to help facilitate T cell receptor signaling activities and the presence of CD3+/CD8+ associated with a good prognosis in colorectal cancer [41]. Therefore, tumor-immune cells with CD3 can increase a cytotoxic immune response in contrast to the presence of THCs with CTLA4.

Furthermore, HLA-DR, CD68, and CD4 proteins were detected for THC regions in liver metastasis. This result suggested that THC can directly express those proteins or recruit other cells that express those cell surface proteins, such as macrophages (CD68), neutrophils (CD68), and helper T cells (CD4), to facilitate and potentiate liver metastasis. Another interesting finding of our study was the pathway enrichment analysis between THCs and tumor-immune cells. Our results showed that many THC pathways are associated with myeloid cells especially neutrophils. This finding suggested that neutrophils can facilitate the transport of colorectal cancer cells to different tissues [42].

In addition, we were able to predict the role of the *CTLA4, CD68*, *CD4* genes in the THC spots for each tissue showing different types of CMS. The identification of CMS helps us to understand and explain the molecular characteristic and function of THCs. Previously, critical information identified that immunosuppressive tumor-associated macrophages were enriched in CMS1 and CMS4 [43]. *CD68* expression was positively correlated with phagocytosis and immune cell infiltration, including dendritic cells, monocytes, macrophages, and neutrophils, but not associated with myeloid-derived suppressor cell infiltration [44]. Anti-CTLA4 immunotherapy can significantly deplete CD68 macrophage in patients with advanced melanoma compared to the untreated sample group [45]. Immunotherapies targeting CTLA4 have been studied in clinical research against advanced CRC [20, 21, 46]. In this study, we found that the *CTLA4*, *CD68,* and *CD4* genes were closely related to THC and CMS1 CRC; therefore, this suggested that CHCs and THCs may act as a new marker of immunotherapies in tumor treatments in the future.

Taken together this finding supported that immune cells and THCs can be different for tumor cell immune escape in each CMS subtype. Our results also supported that bulk transcriptional CMS identification could be a starting point to deepen our knowledge about CRC biology and that its specific immune cell population could support drug discovery and rational combination therapies [47]. In future studies, it will be important to explore the molecular characteristics of THCs along with CMS classification**.**

There are some limitations to this study. Firstly, the GeoMx® spatial proteomic and the Visium CytAssist spatial transcriptomic analysis do not provide single-cell colocalization of THCs and immune cells. This study, the analysis area of the spatial proteomic and the spatial transcriptomic analysis include 20 cells and in the range of 1–10 cells, respectively. The biomarkers which were reported here might resulted from the communication among 1–20 cells. However, many of the immune cell types that were evaluated had shown a cell–cell complex in the tumor. Secondly, the probe set of the Visium CytAssist spatial transcriptome lacked the *HLA* gene family; therefore, *HLA* was not included in spatial transcriptome analysis. We found the upregulation of HLA-DR in the THC region of liver metastasis, but we could not find *HLA* gene in our spatial transcriptome analysis. Finally, a small sample size was one of our limitations. In Table 1, the statistically significant "difference" or "not difference" could be due only by chance. Although the small patient cohort limits our study, we envision that CHCs, THCs and CMSs may potentially inform patient-specific treatment strategies. As CHCs and THCs may act as a new marker of immunotherapies in tumor treatments in the future, we anticipate their application to larger CRC cohorts, paving the way toward personalized oncology.

## Conclusions

This study explored and addressed a metastatic mechanism of THCs and CHCs. Our data may provide insight into possible improvements in clinical practice to combat metastasis in CRC. We suggest that CHCs and THCs with CTLA4 may act as a new marker of immunotherapies in advance CRC treatments in the future [40, 41, 45].

## Supplementary Information

Below is the link to the electronic supplementary material.

Supplementary file1 (XLSX 33 KB)

## Data Availability

Supplementary data are attached in additional files. The data can be obtained in GEO under the accession number: GSE267401 (https://www.ncbi.nlm.nih.gov/geo/query/acc.cgi?acc=GSE267401). The other datasets used and/or analyzed during the current study are available from the corresponding author on reasonable request.
